# Reporting macrolide-resistant *Mycoplasma pneumoniae*: a diagnostic obligation?

**DOI:** 10.1128/jcm.00164-26

**Published:** 2026-06-30

**Authors:** Anisha Misra

**Affiliations:** 1 Department of Pathology and Laboratory Medicine, Cleveland Clinic 161821, Cleveland, Ohio, USA; Cleveland Clinic, Cleveland, Ohio, USA

## Abstract

Macrolide-resistant *Mycoplasma pneumoniae* (MRMP) is increasingly detected worldwide, with increasing prevalence even in the United States. Detection of resistance within *M. pneumoniae* has meaningful clinical and stewardship implications. Resistance is most commonly mediated by point mutations within 23S rRNA and can be reliably identified by molecular assays, not just on isolates butalso on primary respiratory samples like swabs. Multiple studies associate macrolide resistance with prolonged fever, delayed clinical response, increased healthcare utilization, and greater antibiotic exposure due to delay or treatment failure. The detection of resistance can meaningfully influence the clinical course and overall patient outcomes. Though clinical disease is not any more severe in infections with resistant isolates, failure to report may perpetuate unnecessary macrolide use, leading to delay in transition to effective second-line agents (tetracyclines or fluoroquinolones when appropriate), and contribute to selective pressure. Beyond individual patient care, routine reporting provides valuable local epidemiologic data to inform empiric therapy, support outbreak recognition, and guide antimicrobial stewardship initiatives. Even so, a significant clinical gap remains as most commercially available diagnostic assays do not routinely detect or report resistance markers in *Mycoplasma pneumoniae*. Moreover, even among assays designed to detect resistance, performance can vary considerably with reduced sensitivity for organism detection and incomplete identification of key 23S rRNA mutations reported in comparative evaluations, as reported by Hénin N, Silvant A, Gardette M, Balcon C, Guiraud J, Bébéar C, Pereyre S, J Clin Microbiol 64: e0149125, 2026, https://doi.org/10.1128/jcm.01491-25. Focused efforts are, therefore, needed to close existing diagnostic and reporting gaps to ensure resistance detection is more consistently integrated into routine clinical practice.

## INTRODUCTION

*Mycoplasma pneumoniae*, particularly macrolide-resistant strains (MRMP), has transitioned from an emerging threat to an established respiratory pathogen with a significant global presence. In the Western Pacific region, MRMP has a substantially higher prevalence than in other World Health Organization regions, with pooled estimates around 53.4% (1). Reports show a significant increase over the early 2000s through 2019, driven primarily by the A2063G resistance variants, with particularly high rates in countries such as China and Japan (2–6). During epidemic cycles, resistance rates in these countries consistently exceed 80%–90% (6–8).

By contrast, MRMP has been much less common in Europe and the United States, but evidence indicates that resistance is present and increasing in both regions. In Europe, reported macrolide resistance rates have ranged from low single digit values up to 25%, highlighting the variability seen across surveillance systems and methods used (9, 10). In the United States, surveillance studies estimate the frequency of macrolide resistance to be around 8% overall but as high as 15%–21% in southeastern United States (1, 11). Isolated reports document even greater variability during localized outbreaks (11–13).

Following the COVID-19 pandemic, *M. pneumoniae* circulation dropped sharply under widespread nonpharmaceutical interventions. As these measures were lifted, population susceptibility increased, and *M. pneumoniae* resurged in 2023–2024 (14). Recent surveillance data from central Ohio demonstrate this pattern well, with overall MRMP prevalence rates remaining low at around ~2% on average, with evidence that resistance proportions increased over the course of the surge (highest at 4.4%) before returning toward baseline as the overall activity declined (15).

Despite these regional differences, macrolides remain the first-line therapeutic option for *Mycoplasma pneumoniae*, particularly in the pediatric population where alternatives may be limited (16–18). The combination of post-pandemic resurgence, ongoing variability in resistance prevalence, and the emerging upward trend in several regions across the world underscores the importance of sustained molecular surveillance. These data are important for not only recognizing shifts in resistance patterns but also for supporting judicious antimicrobial stewardship and informing empiric treatment decisions in real time.

These observations set the stage for the central issue:

## IS MACROLIDE RESISTANCE IN *MYCOPLASMA PNEUMONIAE* PREDICTABLE?

Yes!

Macrolide resistance in *Mycoplasma pneumoniae* is highly predictable, especially compared with many other bacterial pathogens. In most cases, resistance is due to well-defined point mutations in a highly conserved, single-copy target gene within the 23S rRNA (19, 20). This ability to predict resistance is largely due to the organism’s streamlined genome, roughly ~816 Kb, which, like other mollicutes, has undergone extensive genome reduction. As a result, *Mycoplasma pneumoniae* lacks many of the accessory genetic elements commonly acquired by other bacteria through horizontal gene transfer, including plasmids, transposons, and machinery. This minimalistic genome limits its ability to acquire or accumulate auxiliary resistance mechanisms, making point mutations one of its major drivers of resistance (21). Single-nucleotide substitution in the peptidyl transferase loop, domain V, of the 23S rRNA within the 50S ribosomal unit is sufficient to disrupt antibiotic binding, resulting in a macrolide-resistant phenotype in *Mycoplasma pneumoniae* (22).

Globally, *Mycoplasma pneumoniae* circulates as two genetic lineages, P1 type 1 and P1 type 2. Although these lineages differ epidemiologically and antigenically, macrolide resistance in both correlates with the same underlying mutations, with resistance detected more frequently in P1 type 1 strains (5, 8, 23). Surveillance studies across the globe identify the same substitutions clustered within domain V of the 23S rRNA gene that mark resistance to macrolides (1, 23, 24). The table below summarizes the key mutations, effects on macrolide resistance, and geographic distribution (3, 25–31). For the most part, high-level resistance is defined as minimum-inhibitory concentrations (MICs) of ≥64 mg/L for 14- and 15-membered macrolides and documented association with treatment failure (29, 32). Low-level resistance produces more modest MIC elevations, 1–8 mg/L, and has variable clinical significance (33). This predictable clustering of mutations at a small number of positions enables molecular diagnostics to target just a few nucleotide sites to detect most clinically relevant resistant isolates. Of note, no clinical isolates resistant to tetracyclines or fluoroquinolones have been described to date (33) (Table 1).

**TABLE 1:** Mycoplasma mutations assocaited with altered macrolide binding, predicted resistance phenotype, and reported geographic distribution

Mutation	Effect on macrolide binding	Resistance prediction	Geographical distribution
A2063G^*a*^	Disrupts hydrogen bonding in the macrolide binding site	High	Most common globally
A2064G^*a*^	Alters the adjacent binding pocket structure	High	Worldwide, less frequent than A2063G
A2063C/T	Structural distortion of the binding site	High	Sporadic globally, mostly reported in Asia and Europe
A2067G	Reduced macrolide affinity	Moderate	Rare. Reported in Asia and occasional European cases
C2617G/A	Alters distal binding interactions	Low	Rare case reports in Europe and N. America
C2617T	Alters distal binding interactions	Low to moderate	Rare case reports globally

^*a*^ Double mutations most often involving A2063G and A2064G have been reported, though they occur at a substantially lower frequency than single mutations. When present, double mutants further alter the macrolide-binding site confirmation, decreasing antibiotic affinity and generally conferring even higher resistance than single mutations (24). Additional rare mutations including A2054G, A2058G, A1290G, and C2353T have been reported and appear sporadically in surveillance data (32, 34). Emerging substitutions such as A2063C/T and C2617A/G/T variants have been described in recent surveillance studies; however, these mutations currently remain limited to specific geographical foci (25).

Although point mutations remain the dominant and best-characterized mechanism of macrolide resistance in *M. pneumoniae,* there is emerging evidence that additional pathways may contribute in select cases (35–37). Macrolide-resistant clinical isolates have also been found to carry efflux pump genes msrA/B and mefA, and inhibition with reserprine reduced azithromycin MICs to approximately one-quarter of the baseline value, indicating that efflux activity can partially modulate resistance in these strains (33, 38). Importantly, these observations do not alter the overall predictability of resistance. The resistance landscape in *M. pneumoniae* remains highly stable and overwhelmingly driven by a small defined set of 23S rRNA point mutations, with efflux mechanisms functioning as rare, secondary contributors rather than independent drivers (38–40). Surveillance data from adults in Beijing support this idea, with mutations being the core mechanism for resistance in *Mycoplasma pneumoniae,* and account for the vast majority of resistance, with efflux activity appearing in conjunction in few isolated cases (38).

*M. pneumoniae* culture and culture-based phenotypic susceptibility testing are no longer routinely performed due to the slow and technically demanding growth. Molecular detection has become the standard approach for diagnosis. However, despite the predictable link between genotypic mutations and macrolide resistance, only a limited number of commercially available or widely implemented diagnostic assays explicitly incorporate resistance detection as a part of routine testing. The authors of the study (41) highlight two such assays, TIB Molbiol and Mole Bioscience, and compare their performance characteristics. This remains a critical gap as the strong genotype-phenotype correlation for macrolide resistance makes these mutations highly actionable markers for clinical care, especially when available through rapid molecular platforms.

## DOES MACROLIDE RESISTANCE MEANINGFULLY AFFECT CLINICAL OUTCOMES?

Maybe.

As with most things in microbiology, macrolide resistance in *Mycoplasma pneumoniae* does not have a straightforward clinical signature, and its impact on clinical outcomes remains nuanced. There is no clear association between macrolide resistance detection and increased mortality or higher rates of critical illness as compared with macrolide-susceptible *M. pneumoniae* infections (11). MRMP infections in otherwise healthy children (which are the majority of cases) remain largely self-limited, and serious complications are uncommon regardless of the resistance status (20, 42). However, the absence of increased severity does not mean resistance is clinically irrelevant. Across multiple studies, MRMP is associated with modest but measurable increases in healthcare utilization, demonstrated by effects on the course of illness, including prolonged febrile duration (mean ~ 1.7 days longer) (43–46), longer hospitalization stays on average (mean ~ 1.6 days longer) (42, 47), longer total antibiotic courses (mean ~ 3 additional days) (42, 44), and increased transition to second-line therapy or addition of adjunctive therapeutics (2). These effects highlight an effect on recovery tempo rather than disease severity. Some cohorts have also reported worse radiographic findings possibly due to higher organism burden (42, 48). In other words, macrolide resistance appears to affect the tempo of clinical recovery more consistently than overall severity; patients ultimately recover, but they recover more slowly.

Importantly, these observations also reinforce that not all *Mycoplasma pneumoniae* infections require antibiotic escalation, even when macrolide resistance is detected. In otherwise, immunocompetent patients with mild disease or stable clinical trajectories, *M. pneumoniae* infection may be reasonably managed with symptomatic and supportive care alone, including antipyretics and close clinical observation. Given the self-limited nature of most infections and the modest clinical impact of resistance on disease severity, antibiotic therapy, particularly second-line agents such as tetracyclines or fluoroquinolones, should not be reflexively initiated solely on the basis of a resistant genotype in the absence of clinical deterioration or persistent symptoms.

Moreover, macrolide resistance does not uniformly predict treatment failure. Many patients, children and adults, show improvement despite detection of *in vitro* resistance markers (49). This phenomenon likely reflects a combination of host-directed immune clearance and the anti-inflammatory properties of macrolides (50). Additionally, the observed variability in treatment efficacy of macrolides in MRMPs may be due to country and institutional practices (51). For example, in South Korea, adjunctive corticosteroids are often used early to mitigate the effect of resistance on the clinical response (52), whereas in Japan, steroids are reserved for refractory cases only (53). On the other hand, in China, where resistance rates are significantly higher, treatment with just macrolides has been associated with more extrapulmonary complications (43) suggesting that the clinical impact of resistance may be amplified in settings with elevated transmission dynamics and high organism burden. Additionally, molecular detection of MRMP may reflect asymptomatic carriage or reflect nonviable genomic detection after true infection, further complicating the interpretation of resistance in relation to the clinical response (54). These findings highlight that no one factor, which includes resistance detection, dictates recovery, and host factors, adjunctive therapies, and local management strategies play a role in influencing outcomes.

What does this mean for whether resistance should be reported routinely or how results should be interpreted and acted upon clinically? Taken together, these findings underscore that macrolide resistance should not be interpreted in isolation or as an automatic trigger for therapy escalation nor should it be treated as a marker for impending complications. Instead, resistance functions as a modifier of expected response kinetics rather than a predictor of severe disease. Knowledge of macrolide resistance becomes most clinically meaningful in the setting of delayed recovery. For patients (particularly children) who remain febrile or symptomatic beyond 48–72 h of macrolide therapy, the presence of a known resistance mutation provides context. It allows clinicians to differentiate expected slow response from true treatment nonresponse and to initiate transition to second-line therapy more confidently. This interpretive guidance then can shorten the time to effective therapy, especially during high-burden seasons or epidemics. The value in reporting macrolide resistance, therefore, is not as a prognostic marker of severe disease but as a practical modifier of the expected treatment response that supports timely, targeted clinical interventions when recovery does not follow the anticipated course.

In practice, resistance reporting would be most helpful when fevers or symptoms persist beyond 48–72 h, particularly in the pediatric population where macrolides are first-line therapy and alternatives carry age-specific considerations. With molecular markers of resistance, resistance can reliably be identified within this early window, providing actionable information that can shorten the time to effective second-line therapy if no improvements are seen within 72 h. Reporting resistance provides context of expected treatment response, facilitates timely and precise interventions only when recovery is delayed, and improves antibiotic stewardship goals by guiding escalation rather than empiric overuse. Resistance reporting, therefore, supports targeted rather than reflexive decision-making. These clinical nuances, however, raise another important question:

## IF MACROLIDE RESISTANCE IS REPORTED IN MRMP, DOES THAT RISK UNNECESSARY ANTIBIOTIC ESCALATION?

Possibly, but only if resistance is evaluated in isolation of the clinical context.

Real-world prescribing data suggest that reporting macrolide resistance does not, by itself, mandate immediate therapy escalation. Prescribing decisions are still anchored to the clinical trajectory (such as persistent fever, nonresponse, or hospitalization with a severe case). In a retrospective study of 247 hospitalized children with *Mycoplasma pneumoniae* pneumonia, with 66 macrolide-resistant and 181 macrolide-susceptible cases, investigators examined antibiotic selection, adjunctive therapy, and healthcare utilization (55). In individual retrospective cohorts, including a study of hospitalized children, length of stay did not differ significantly between MRMP and macrolide-susceptible cases, underscoring the heterogeneity in observed outcomes across study designs and clinical settings. However, MRMP was associated with broader antimicrobial use, and patients were more likely to receive doxycycline (approximately 71%), whereas macrolide monotherapy remained the most common regimen for MSMPP (34.5%) (55). Taken together, these findings suggest that while macrolide resistance is associated with modest delays in clinical recovery at the population level, its impact on hospitalization metrics such as length of stay is heavily influenced by patient selection, illness severity, and institutional management practices.

Interestingly, ~30% MRMP pneumonia patients did not receive doxycycline, indicating that resistance detection did not uniformly mandate immediate transition to second-line therapy. Moreover, the timing of doxycycline initiation was not clearly linked to the report of resistance. Escalation decisions most likely reflected the hospitalized nature of these patients and the broader clinical context, such as persistent fever or perceived severity (rather than resistance reporting alone) (32, 55). Similarly, combination therapy was also more common in MRMP patients, with 29% receiving doxycycline and piperacillin-tazobactam, despite beta-lactams lacking activity against *M. pneumoniae,* suggesting that escalation patterns often reflect clinical uncertainty or concern for co-infection rather than resistance alone (55).

This framework is consistent with broader clinical microbiology practices. Laboratories routinely report resistance in culturable macrolide-resistant *Streptococcus pneumoniae*, a pathogen with similar clinical presentation and often self-limited disease, and treatment decisions remain fundamentally clinical (56–58). Resistance reporting contextualizes management rather than dictating it. Recent expert guidance developed through a structured Delhi methodology echoes this approach, emphasizing that MRMP should inform management primarily when the clinical response is inadequate (32). Macrolides continue to remain the first-line treatment. Transition to tetracyclines or fluoroquinolones or other adjunct therapy is recommended primarily in the setting of persistent fever and clinical nonresponsiveness rather than on the basis of the resistant genotype (32). This reliance on the clinical trajectory largely reflects the absence of timely, objective laboratory data on macrolide resistance rather than a lack of clinical utility of resistance information. In the setting of limited access to rapid genotypic antimicrobial resistance testing, healthcare systems have developed management strategies that depend on the observed clinical response; however, the availability of reliable resistance assays would be expected to meaningfully influence prescribing decisions by enabling earlier, evidence-based differentiation between the expected delayed response and true microbiologic treatment failure. Assays such as those described in the study by Henin et al. have the potential to shift this paradigm by making resistance information available within clinically relevant timeframes, thereby complementing, rather than replacing, clinical assessment and supporting more informed targeted therapy decisions.

A key modifier of this discussion is the ability to detect resistance by rapid molecular assays. Unlike culture-based susceptibility testing, which is impractical for *M. pneumoniae* due to the slow and insensitive growth, molecular detection can identify both the pathogen and resistance mutations within clinically actionable timeframes. Studies on pediatric cohorts demonstrate that early molecular identification of macrolide resistance mutations enables clinicians to distinguish the expected delay response from true microbiologic failure and tailor therapy more effectively (20, 51, 59). To this end, the stewardship value of resistance reporting is closely related to turnaround time; rapid molecular detection transforms resistance from retrospective epidemiologic information into prospective clinical guidance.

Not every resistance result necessitates a change in therapy, and clinical laboratories do not withhold antimicrobial resistance values to control prescribing behavior. The value of reporting resistance, especially for *Mycoplasma pneumoniae,* is to inform decision-making when the clinical response is uncertain. Without resistance reporting, the risk is prolonged ineffective macrolide therapy in sick patients or escalating empirically to broader-spectrum agents. What it does highlight, from a stewardship perspective, is the need to report resistance with interpretive context, particularly in pediatrics, to limit unnecessary escalations, while enabling timely transition to effective therapy when warranted.

However, when initial therapy is chosen empirically before patient-specific results are known, population-level resistance data can be extremely valuable. Rising macrolide resistance during an outbreak or epidemic can shift the probability that first-line macrolides will be effective, influencing empiric prescribing choices and highlighting the broader role of surveillance (12, 15).

## WHY IS RESISTANCE TESTING NOT ROUTINELY PERFORMED TODAY?

Despite the predictable molecular basis of macrolide resistance in *Mycoplasma pneumoniae* and its demonstrated interpretive value in selected clinical scenarios, routine resistance testing remains uncommon in most clinical settings. This gap reflects practical implementation barriers rather than a lack of clinical relevance. At present, only a limited number of commercial assays incorporate resistance detection, and access in the United States is largely restricted to reference or academic laboratories, where longer turnaround times reduce the immediate impact on clinical decision-making. In addition, some assays capable of resistance detection exhibit reduced sensitivity for organism detection or incomplete coverage of clinically relevant resistance mutations, including substitutions at position 2067 of the 23S rRNA gene. Practical considerations further constrain adoption, including higher testing costs, batch-based workflows in reference laboratories, and logistical delays that limit the availability of resistance results within the early reassessment window when they are most actionable. Ongoing development and implementation of additional molecular assays such as those recently described by Henin et al. (41) have the potential to lower these barriers by expanding mutation coverage, improving analytic performance, and facilitating integration of resistance detection into routine diagnostic workflows.

## HOW DOES POPULATION-LEVEL RESISTANCE SURVEILLANCE COMPLEMENT INDIVIDUAL PATIENT MANAGEMENT?

Systematic surveillance of resistant trends informs empiric therapy and stewardship strategies.

While individual resistance results primarily refine bedside decision-making, population-level data address a different challenge. We know resistance in *Mycoplasma pneumoniae* is not static. The organism follows distinct seasonal patterns and multiyear epidemic cycles. MRMP prevalence can shift rapidly, particularly during surges (12, 15). Without systematic testing and reporting, these fluctuations remain largely invisible, leaving clinicians to make empiric decisions based on outdated or incomplete assumptions. Population-level surveillance provides the broader context needed to anticipate these shifts, refine empiric therapy, and guide public health response.

Although the genome of *M. pneumoniae* is evolutionarily conserved, its epidemiology is distinctly cyclical. Epidemic peaks occur every 3–7 years and may persist for 1–2 years, as observed in Asia and Europe prior to the COVID pandemic (60–63). Importantly, macrolide resistance rates tend to rise during these epidemic waves, often coinciding with the expansion of dominant clones. For instance, during epidemic periods in Japan and South Korea, there was a dramatic increase in macrolide resistance, with rates exceeding 80% in some regions (64–66). Similar dynamics were reported in China, where epidemic surges have paralleled sharp increases in resistance prevalence (3, 7, 63, 67).

Genotypic shifts frequently accompany these epidemic waves. *Mycoplasma pneumoniae* strains circulate primarily and are classified as P1 type 1 and P1 type 2 lineages based on the variation in the major adhesin gene (MPN141) with multiple subvariants within each lineage (68–72). Epidemiology studies across various sites have shown that P1 lineages are associated with a higher resistance profile than P2 lineages (23, 63, 73). During the 2023 surge of *Mycoplasma pneumoniae* in China, the P1-1 genotype predominated and macrolide resistance was detected in 97.1% of isolates (63). In contrast, only 20% of the P1-2 gene subtype strains had a resistant genotype. Similar patterns have been reported elsewhere; between 2017 and 2019, a Taiwanese cohort documented that 77% of pediatric *M. pneumoniae* cases were macrolide-resistant, largely driven by the clonal expansion of sequence type 3 and its single locus variant ST17, both belonging to the P1-1 lineage (47, 64). Whole-genome sequencing revealed recombination events in the non-P1 adhesin genes involving repetitive elements (RepMP regions), suggesting that genomic plasticity in adhesins contributes to immune evasion and allows resistant clones to persist and circulate (69).

Even in regions with overall low macrolide resistance rates such as the United States, similar epidemic-associated increases and clonal clusters have been documented in regional outbreaks (11, 15, 68). These outbreaks reinforce that MRMP does not emerge randomly but often expands through defined sequence types under favorable epidemiological conditions.

Geographical differences in macrolide resistance prevalence may also be explained by antimicrobial exposure patterns in these areas (57, 74–76). Trends in lower resistance prevalence are supported by nationwide data showing changes in prescribing behavior, specifically driven by stewardship and policy efforts. In Japan, the introduction of financial incentives for clinicians to avoid unnecessary antibiotic prescriptions was associated with a 17%–22% reduction in over-antibiotic use without increasing adverse outcomes such as hospitalizations (77). Reviews of interrupted time-series studies and current antibiotic use in Japan also highlight that targeted efforts to reduce unnecessary use, including guidelines dissemination and stewardship initiatives, correlate with decreasing trends in prescriptions for common infections (78–81). Specifically, for *Mycoplasma pneumoniae*, the temporary reduction in resistance in Japan between 2018 and 2023, from 81.8% to 14%–33%, coincided with decreased macrolide use and the availability of tosufloxacin, a quinolone used effectively in children to limit MRMP transmission (82–84). In contrast, high resistance rates in neighboring countries like China and South Korea correspond with widespread macrolide use and limited access to alternative agents (82). Similarly, European countries such as Germany maintain low MRMP prevalence, likely due to low macrolide prescribing (61, 85), illustrating how population-level data, antimicrobial use, and resistance ecology are tightly connected.

These observations highlight the value of systemic, real-time resistance surveillance. Surveillance does more than just provide retrospective prevalence estimates; it offers actionable feedback that can guide empiric prescribing, signal when alternative therapies may be warranted, and help blunt the spread and clonal amplification of resistant strains. Without continuous ongoing monitoring, shifts in resistance often go unnoticed until they are investigated due to widespread treatment failure (12). Real-time assessment and reporting allow resistance trends to inform population-level stewardship and empiric therapy decisions, helping prevent the expansion and dissemination of highly resistant genotypes during epidemic cycles.

## SHOULD RESISTANCE REPORTING BE UNIVERSAL OR CONDITIONAL?

Universal, ideally but conditional in practice due to real-world limitations.

Macrolide resistance in *Mycoplasma pneumoniae* is no longer a rare or theoretical microbiologic phenomenon. Given its predictable molecular basis, measurable impact on treatment response, and influence on antibiotic use, failure to report resistance constitutes a missed opportunity for both individualized care and broader antimicrobial stewardship efforts. Ideally, resistance would always be reported given its implications at both an individual and population level.

In practice, routine reporting of resistance in *Mycoplasma pneumoniae* remains uneven. Universal reporting is often limited by access to validated molecular assays, the availability of regulatory approved platforms, and resource limitations in many clinical laboratories. At present, for the most part in the United States, resistance testing limited to large reference or academic centers, where longer turnaround times can diminish the immediate clinical impact of results (11, 13). Expanding assay availability and integrating resistance detection into existing diagnostic workflows is, therefore, critical. Melt curve-based PCR assays are particularly practical for mutation detection; they are rapid, compatible with many molecular platforms already in routine use, and are capable of detecting key macrolide resistance mutations directly from the patient samples. In practice, this enables same-day linkage of organism detection with resistance calls, making results clinically actionable within that reassessment window to guide therapy changes. Integrating any PCR-based mutation detection as a reflex add-on to positive *M. pneumoniae* tests converts resistance reporting from a retrospective, could-have, should-have clinical conundrum to real-time clinical guidance.

However, based on real-world constraints, it may be more practical to take a pragmatic tiered approach to resistance testing and reporting for *Mycoplasma pneumoniae*. In high-prevalence settings, during known outbreaks or in pediatric population where macrolides remain the first-line therapy, incorporating routine resistance testing alongside detection provides the most timely clinical and stewardship value. In lower-prevalence settings or resource-limited settings, resistance testing can be prioritized for patients who fail to improve after 48–72 h of macrolide therapy, those requiring hospitalization, or those demonstrating radiographic worsening despite treatment. Under these circumstances, even when testing is only available through reference laboratories with longer turnaround times, results remain valuable for guiding therapy adjustments, guiding local stewardship policies, and improving the accuracy of regional surveillance data.

Ultimately, reporting macrolide resistance should be considered a diagnostic obligation wherever molecular capacity exists and expanded wherever feasible. By integrating accessible, validated reflex resistance testing into routine workflows, pairing detection with structured interpretive guidance, and applying practical decision algorithms based on local prevalence and patient risk factors, resistance reporting can shift from a retrospective epidemiological exercise to a tangible, actionable tool. This approach ensures that clinicians have all the tools available to them to make informed therapeutic decisions. Additionally, real-time resistance data would factor into antibiotic stewardship, and public health authorities can respond proactively to emerging resistance patterns.
